# Implementing Digital Respiratory Technologies for People With Respiratory Conditions: Scoping Review

**DOI:** 10.2196/88325

**Published:** 2026-06-16

**Authors:** Io Chi-Yan Hui, Kathleena Condon, Shailesh Kolekar, Nicola J Roberts, Katherina Bernadette Sreter, Sami O Simons, Carlos Figueiredo, Zoe McKeough, Hani Salim, Aleksandra Gawlik-Lipinski, Apolline Gonsard, Ayşe Önal Aral, Anna Vanoverschelde, Matthew Armstrong, Dario Kohlbrenner, Cátia Paixão, Patrick Stafler, Efthymia Papadopoulou, Adrian Paul Jaravata Rabe, Milan Mohammad, Izolde Bouloukaki, Shirley Quach, Georgios Kaltsakas, Kate Loveys, Tonje Reier-Nilsen, Anthony Paulo Sunjaya, Paul Robinson, Michaela Senek, Amy Hai Yan Chan, Hilary Pinnock

**Affiliations:** 1 School of Population Health Sciences, Usher Institute, The University of Edinburgh, 5 Little France Rd, Edinburgh, EH16 4UX, United Kingdom, 44 0131 651 7869; 2 Faculty of Health, Medicine and Behavioural Sciences, University of Queensland, Brisbane, Queensland, Australia; 3 Department of Medicine Helsingborg Hospital, Lund University, Region Skane, Sweden; 4 School of Health and Social Care, Edinburgh Napier University, Edinburgh, United Kingdom; 5 Department of Pulmonology, University Hospital Centre "Sestre Milosrdnice", Zagreb, Croatia; 6 NUTRIM Institute for Nutrition and Translational Research in Metabolism, Faculty of Health, Medicine and Life Sciences, Maastricht University Medical Center+, Maastricht, The Netherlands; 7 Hospital Santa Marta, ULS São José, Lisboa, Portugal; 8 Sydney School of Health Sciences, University of Sydney, Sydney, NSW, Australia; 9 Department of Family Medicine, Universiti Putra Malaysia, Serdang, Selangor, Malaysia; 10 Department of Respiratory Sciences, University of Leicester, Leicester, United Kingdom; 11 University Hospital Necker-Enfants Malades, AP-HP, Paris, France; 12 Pulmonary Diseases Clinic, Ankara Gölbaşı State Hospital, Gölbaşı/Ankara, Turkey; 13 General Hospital Zeno, Knokke-Heist, Belgium; 14 Department of Sport and Exercise Sciences, University of Durham, Durham, United Kingdom; 15 Epidemiology, Biostatistics, and Prevention Institute, University of Zurich, Zurich, Switzerland; 16 Respiratory Research and Rehabilitation Laboratory (Lab3R), School of Health Sciences (ESSUA) and Institute of Biomedicine (iBiMED), University of Aveiro, Aveiro, Portugal; 17 Schneider Children's Medical Center of Israel and Gray Faculty of Life Sciences, Tel Aviv University, Petah Tikva, Israel; 18 Department of Respiratory Failure, G. Papanikolaou General Hospital of Thessaloniki, Thessaloniki, Greece; 19 School of Public Health, Imperial College London, London, United Kingdom; 20 Centre for Physical Activity Research, Copenhagen University Hospital, Copenhagen, Hovedstaden, Denmark; 21 Department of Social Medicine, School of Medicine, University of Crete, Heraklion, Crete, Greece; 22 School of Rehabilitation Sciences, Faculty of Health Sciences, McMaster University, Hamilton, ON, Canada; 23 Centre for Human & Applied Physiological Sciences, King's College London, London, United Kingdom; 24 Department of Paediatrics: Child and Youth Health, School of Medicine, the University of Auckland, Auckland, New Zealand; 25 Oslo Sports Trauma Research Center, Insitute of Sports Medicine, Norwegian School of Sport Sciences, Oslo, Norway; 26 School of Population Health, University of New South Wales, Sydney, NSW, Australia; 27 School of Medicine& Population Health, University of Sheffield, Sheffield, United Kingdom; 28 School of Pharmacy, The University of Auckland, Auckland, New Zealand

**Keywords:** digital health, respiratory, artificial intelligence, implementation, review

## Abstract

**Background:**

Digital health offers opportunities for safe, equitable, and accessible care, and its integration into respiratory care is a strategic priority for the European Respiratory Society. However, sustainable implementation remains complex, and guidance for health care systems is limited.

**Objective:**

This study aimed to undertake a scoping review of the published initiatives that have implemented digital respiratory technologies into real-world routine clinical practice over the past decade, identify the technologies used, implementation strategies used, the challenges and supports they encountered, and the lessons they reported for making care more equitable, strengthening patient-professional relationships, improving the patient journey, and reducing environmental impact.

**Methods:**

Following Arksey and O’Malley’s methodology, we searched ten databases (December 2013‐2023 [updated April 2025 and February 2026]): MEDLINE, Embase, CINAHL, PsycINFO, Cochrane Library, Web of Science, Scopus, IEEE Xplore, CABI Global Health, and WHO Medicus; and used key domains in the commonly used implementation frameworks such as the Consolidated Framework for Implementation Research (CFIR), Nonadaptation, Abandonment, and Challenges to the Scale-up, Spread, and Sustainability of Health and Care Technologies (NASSS), and the Reach, Effectiveness, Adoption, Implementation, and Maintenance (RE-AIM) framework to categorize results and understand methodologies used. As a scoping review, we mapped the available evidence, rather than synthesizing outcomes, appraising study quality, or estimating effectiveness. To broaden coverage and strengthen interpretation, we crowdsourced additional studies and sought feedback on our preliminary findings from a network of respiratory experts across 17 countries.

**Results:**

Overall, 24,672 studies were identified; after deduplication, 14,811 were screened; 84 studies from 31 countries were included in the final review. The digital respiratory technologies comprised apps, platforms, chatbots, and smart devices. Reported technological functionalities encompassed remote consultation, clinician monitoring, video directly observed therapy, remote rehabilitation training, self-management support, education, monitoring medication adherence, and a school-based remote clinic. CFIR, RE-AIM, and the plan-do-study-act (PDSA) cycle were the most widely used frameworks. Successful implementation used simple technologies that fitted existing workflows and avoided additional workload. Co-development and trust-building with end-users influenced motivation and adoption, while leadership, team cohesion, and communication facilitated success. Barriers included insufficient resources, poor interoperability, lack of funding and reimbursement, and limited technical support.

**Conclusions:**

This scoping review provides a cross-condition review of digital respiratory technologies implemented in routine clinical practice. Unlike previous disease-specific or experimental-focused reviews, our innovative approach used established implementation Theories, Models, and Frameworks (TMFs) to identify shared barriers and enablers across diverse populations and health care systems. We summarize key implementation domains in state-of-the-art digital respiratory care and identify major gaps related to health equity, patient–clinician trust, continuity of support, and environmental sustainability. These findings emphasize the value of using implementation TMFs for scaling effective, patient-centered digital respiratory care in real-world settings.

## Introduction

**Figure 1.**
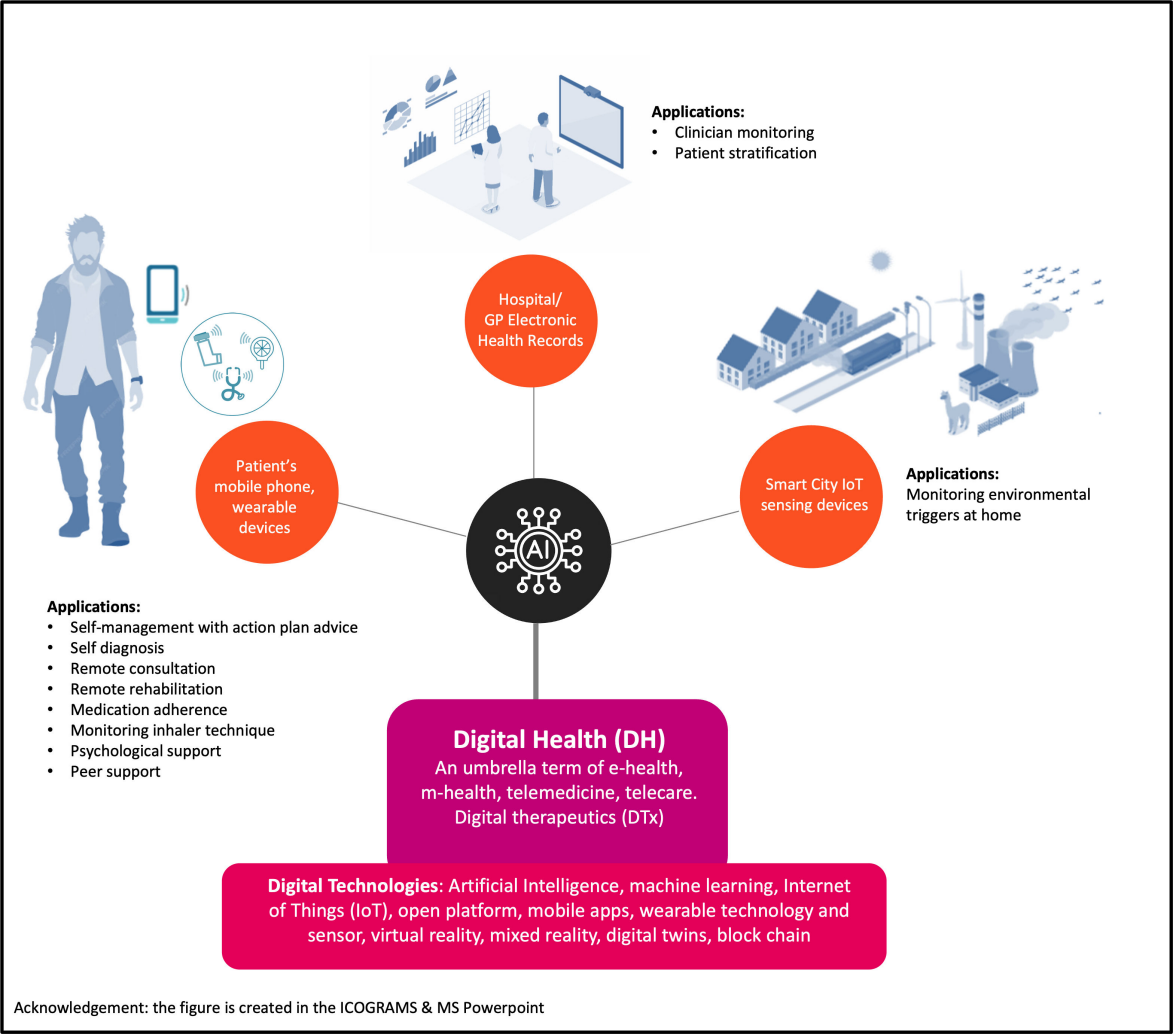
Digital respiratory health landscape. Image created using ICOGRAMS EDUCATION, under CC-BY license [8,9]. AI: artificial intelligence; GP: general practitioner.

Chronic respiratory diseases affect more than 545 million people worldwide, have a significant impact on individuals’ quality of life, and are a major burden to health care systems [1]. Digital health tools that can contribute to regular monitoring of respiratory conditions are prone to exacerbation and support better health outcomes by enabling timely interventions. Conventionally, telemedicine, telecare, e-health, and mobile health have been defined as the use of electronic means, such as information and communication technologies, or mobile devices and remote sensors, to deliver health services [2,3]. Digital therapeutics are “interventions that are driven by high-quality software programs to treat, manage, or prevent a disease or disorder*”* [4]. Digital respiratory health is an umbrella term for all these modalities, potentially incorporating artificial intelligence (AI), to support routine respiratory care [5] (Figure 1). Apps, platforms, and smart wearable or portable devices can be interconnected to support diagnosis, underpin personalized medicine, and enable self-management. Real-time video facilitates clinician-supervised remote rehabilitation by enabling health care professionals to guide patients through prescribed exercises within their homes [6]. It also allows clinicians to directly observe patients’ medication adherence and inhaler technique, thereby supporting more accurate assessment and timely intervention. By integrating environmental data and using AI as the “brain,” the system can advise on actions to prevent exacerbations and hospitalizations. Routine data collected from these tools aids interpretation of medical images and public health decisions [7].

The global COVID-19 pandemic accelerated adoption of digital health technologies, enabling rapid deployment potential [5-7]. However, integrating digital health technologies into routine care is complex. Short-term success does not guarantee long-term sustainability. To address this challenge, implementation Theories, Models, and Frameworks (TMFs) were developed. They provide considerations (“domains”) and a systematic approach to guide planning and evaluation of the digitization process, helping organizations to identify barriers and facilitators for successful implementation. They also share similar descriptions of the domains, but terminologies vary. Widely used TMFs are Consolidated Framework for Implementation Research (CFIR), Nonadaptation, Abandonment, and Challenges to the Scale-up, Spread, and Sustainability of Health and Care Technologies (NASSS), Reach, Effectiveness, Adoption, Implementation, and Maintenance (RE-AIM), Exploration, Preparation, Implementation, and Sustainment (EPIS), Unified Theory of Acceptance and Use of Technology (UTAUT), Technology Acceptance Model (TAM), and Theoretical Domains Framework [10]. CFIR and NASSS framework are determinant frameworks that delineate categories and specific domains that function as either barriers or facilitators, thereby influencing the outcomes of implementation processes [11,12]. RE-AIM is an evaluation framework that provides specific aspects of domains that can be evaluated to determine the success of an implementation [13]. EPIS is a process model and determinant framework that outlines the domains and sequential steps involved in translating interventions into routine practice [14]. UTAUT and TAM are technology-focused models. They conceptualize technology adoption as domains of perceived usefulness and perceived ease of use [15,16]. The UTAUT extends this foundation by integrating 8 prior acceptance frameworks and identifying 4 primary determinants—performance expectancy, effort expectancy, social influence, and facilitating conditions—moderated by user characteristics such as age and experience. Theoretical domains framework is a classic theory that can be applied to enhance understanding and/or explanation of various aspects of implementation [17]. Of these well-known implementation TMFs, CFIR is the most frequently used TMF. CFIR, NASSS, and RE-AIM are more widely used TMFs as they are relatively comprehensive, covering at least 2 phases of implementation (planning, early implementation, and late implementation) [18]. Despite the similarity in the coverage of the domains, the language and glossary are not standardized. Lack of harmonization of domains across borders and sectors [19] obstructs the digital transformation of health care, preventing it from providing safe, fair, and accessible care for everyone [20].

The World Health Organization (WHO) considers digital transformation to be essential for achieving universal health coverage, the EU (European Union) endorses implementation of technological advances to improve patient outcomes and promote patient-centered care, and adoption of digital health is a strategic priority for the European Respiratory Society (ERS) [21,22]. However, only a minority of digital health studies progress beyond the pilot or local intervention stage [23], and harmonization across borders and sectors is rare [19], with the welcome exception of the European Health Data Space, which has been greeted by the ERS as an opportunity to push forward health care, research, and policymaking [24,25].

Digital respiratory technologies are increasingly being adopted across a wide range of respiratory conditions and in diverse health systems. Exploring and adapting to clinical and socioeconomic context is crucial for successful local implementation, though shared barriers and enablers can provide useful learnings that help shape the feasibility and uptake of digital interventions [1,4]. Adopting a global perspective enables the learnings from successful implementation efforts to inform less developed technological initiatives. Using TMFs can support this process by ensuring the challenges are comprehensively considered; this is the underlying rationale for this global scoping review.

The CONNECT Clinical Research Collaboration (CRC) was launched by the ERS in 2023 with the goal of addressing the overarching challenges of whole systems implementation of respiratory digital health care. The core, overarching objective of CONNECT is to bridge the gap between existing disease- or location-focused digital respiratory health initiatives and sustainable, equitable, connected implementation into clinical respiratory practice in diverse health care systems [26]. CONNECT has 5 objectives, one of which focuses on systematically reviewing existing digital respiratory health care embedded in routine clinical practice. In this scoping review (WPIII), we explored published initiatives that integrated and implemented digital respiratory technologies into routine clinical workflows. Our objectives were to scope and describe:

1. Characteristics of respiratory digital health initiatives implemented in routine clinical practice in the last 10 years.
2. TMFs are used to develop and evaluate the implementation strategies.
3. Barriers and enablers to implementation.

Insights relevant to reducing inequity, enhancing patient and professional relationships, supporting the patient journey, and reducing adverse environmental impact.

## Methods

### Overview

We followed Arksey and O’Malley’s methodology for scoping reviews [27] and used Covidence software to manage the review process, including to de-duplicate the studies [28]. We used the PRISMA-ScR (Preferred Reporting Items for Systematic Reviews and Meta-Analyses extension for Scoping Reviews) to guide reporting [29] and PRISMA-S to report the review methodologies and findings [30].

### Published Protocol

Detailed methodology is described below with additional details available in our published protocol [31]. There were no deviations from the protocol.

### Information Sources

We included ten databases (MEDLINE, Embase, CINAHL, PsycINFO, Cochrane Library, Web of Science, Scopus, IEEE Xplore, CABI Global Health, and WHO Medicus). We browsed reference lists manually with forward searches on included studies using the International Statistical Institute Proceedings. To ensure robust representation of research from low- and middle-income countries, we incorporated the low- and middle-income country (LMIC)–focused databases CABI Global Health and WHO Medicus. In addition, we consulted 28 colleagues in the CONNECT group to identify the missing studies in their networks (see Peer Review and consultation exercise section below for details).

### Search Strategy

The search strategy was newly developed for this review to reflect its defined scope and objectives. We developed this search strategy with the Academic Support Librarian at the University of Edinburgh, used the key search terms of “respiratory condition” AND “digital technology” AND “implementation” to search ten databases over a 10-year period from December 12, 2013, to December 12, 2023 (update search performed on April 4, 2025, and February 7, 2026). Given the substantial number of studies, we applied a time limit filter to capture only those falling within the defined search window. In the update searches, we re-ran the same search terms and methods during the update search to identify the newly published studies. See Multimedia Appendix 1 for detailed search strategy.

### Eligibility Criteria

We included any digital health intervention implemented to support routine patient care. Patient care included but was not limited to digital support for diagnosis, self-management, monitoring, medication adherence and compliance, education, psychological support, social support, remote consultation, or health professional-facing interventions. For example, a decision support system that was designed to support delivery of care was included, but an administrative system for scheduling appointments would be excluded.

Implementation studies are not always accurately indexed, so we defined the key features of “implementation” to guide the selection process.

The inclusion and exclusion criteria are outlined in Table 1. See Multimedia Appendix 2 and our published protocol [24] for further details of the criteria and definitions.

**Table 1.** Inclusion and exclusion criteria.

Criterion	Definition	Operationalization and examples
Inclusion criteria		
Population	Patients with any respiratory conditions (short or long-term) of all ages	Where there was doubt about whether a condition was “respiratory,” we included those conditions covered by the Assemblies and Groups of the ERS^a^
Intervention	The intervention should be delivered within the existing service and available to all clinically-eligible individuals (specifically not only to people who consent to the research)	Existing staff may be upskilled for the purpose, but interventions that require additional staff or resources for day-to-day delivery are unlikely to be sustained beyond the end of the project and were therefore excluded
Setting	Any health care setting was eligible	—^b^
Comparator	Typically usual care	Many studies used before-and-after observational designs
Outcomes	Outcomes should reflect the uptake and impact of the intervention within the population	for example, using routine data
Duration:	Any duration	We did not specify a duration for the evaluation, but only included studies in which the intervention was (or was intended to be) embedded for the long term in routine care
Study design	All study types	No geographical or language restrictions
Location	Any location	We did not exclude by language. CONNECT is a global network with members fluent in most major languages
Exclusion criteria		
Administrative only	Digital health interventions that did not directly involve patient care	for example, interventions such as workflow management, appointments, or triage systems with exclusively administrative purposes
Education	Health professional education	for example, online conferences and online courses
Pandemic management	Population-level COVID-19 initiatives, such as contact tracing or vaccination programs	COVID-19 was considered as a respiratory condition when studies focused on individual-level care, such as hospital-at-home programs or management of COVID-19 in people with chronic respiratory disease
Effectiveness research	Randomized controlled trials conducted to test the effectiveness of the intervention	for example, those requiring participant consent process to individual randomization) were excluded as our interest is in implementation in routine clinical care
Research types		
Conference abstracts	Potentially relevant abstracts were tagged and monitored for subsequent publications.	—
Review articles	Reviews and other relevant papers that did not meet inclusion criteria were retained for background reading and reference checking	—
Gray literature	We did not search for gray literature	—

a ERS: European Respiratory Society.

b Not applicable.

### Selection Process

This review was conducted by twenty-seven volunteers from the ERS CONNECT CRC, with a few hours funded by CONNECT for a post-doctoral researcher (ICYH) to provide training, oversight, and ensure quality control. Independent duplicate screening was carried out by the trained volunteers, with disagreements resolved by ICYH, AHYC, and HP in discussion with the 6 volunteers (ZM, AOA, NJR, KBS, SOS, and CF) who reviewed most studies (see protocol for further description of this process [31]). Divergent interpretations (eg, of implementation-related concepts, and which exclusion criterion took precedence) were clarified in discussion, and we subsequently achieved 100% agreement.

### Data Charting, Extraction, and Data Items

Using a similar model of selected volunteers (AOA, SOS, SK, and CF) overseen by the lead author (ICYH, with AC and HP), data extraction was conducted in duplicate, with a final check on accuracy by ICYH. Data extraction was completed using a piloted, refined data extraction sheet with headings reflecting the four objectives (see Multimedia Appendix 3 for the full data extraction table).

#### Collating, Summarising and Reporting the Results

We used a PRISMA (Preferred Reporting Items for Systematic Reviews and Meta-Analyses) diagram to report the selection process [29,30] and collated a narrative summary of qualitative data with charted results from the quantitative data to address the review objectives:

1. Objective 1: we used a bubble plot of the technology components and their associated respiratory conditions to visualize the features integrated into digital health initiatives across various respiratory conditions.
2. Objective 2: we identified the commonly used frameworks (see description in the introduction) and their usage in different countries and income groups. Initially, we used NASSS domains (Condition Complexities, Technology, Value Proposition, Adopters, Organization, Wider System, Embedding, and Adaptation Over Time) to categorize reporting, subsequently integrating features from CFIR and RE-AIM to develop an enriched model that comprehensively captures the complex dynamics influencing the implementation of digital technology within routine clinical practice.
3. Objectives 3 and 4: we used narrative synthesis to categorize the implementation barriers and enablers identified by the authors, and their insights related to health inequities, patient–professional relationships, patient care pathways, and environmental impacts.

#### Peer Review and Consultation Exercise

We engaged with colleagues from the CONNECT CRC collaboration from the outset and maintained ongoing interaction throughout the review process. This group includes academic researchers, clinicians with experience in implementing digital respiratory health technologies, and collaborators with technological expertise. Representing 17 countries across both high-income and low- and middle-income settings, 28 colleagues provided diverse perspectives on the review process. We conducted online workshops at key stages of the review.

1. Beginning of the review: to explore topical themes and practical challenges and refine research questions.
2. Title and abstract, full text screening stages: to fine-tune the inclusion criteria, exclusion criteria, and discuss selection conflicts, crowdsourced potential studies to identify gaps in the included studies and suggest additional papers for consideration.
3. Data charting and collation stages: we recorded a short video summarizing preliminary findings and used an online memo board to gather asynchronous feedback, facilitating engagement across different time zones. The feedback was collected in parallel with the discussions after our abstract presentation at the ERS Congress to finalize the findings and the practical interpretation.

## Results

### Overview

**Figure 2.**
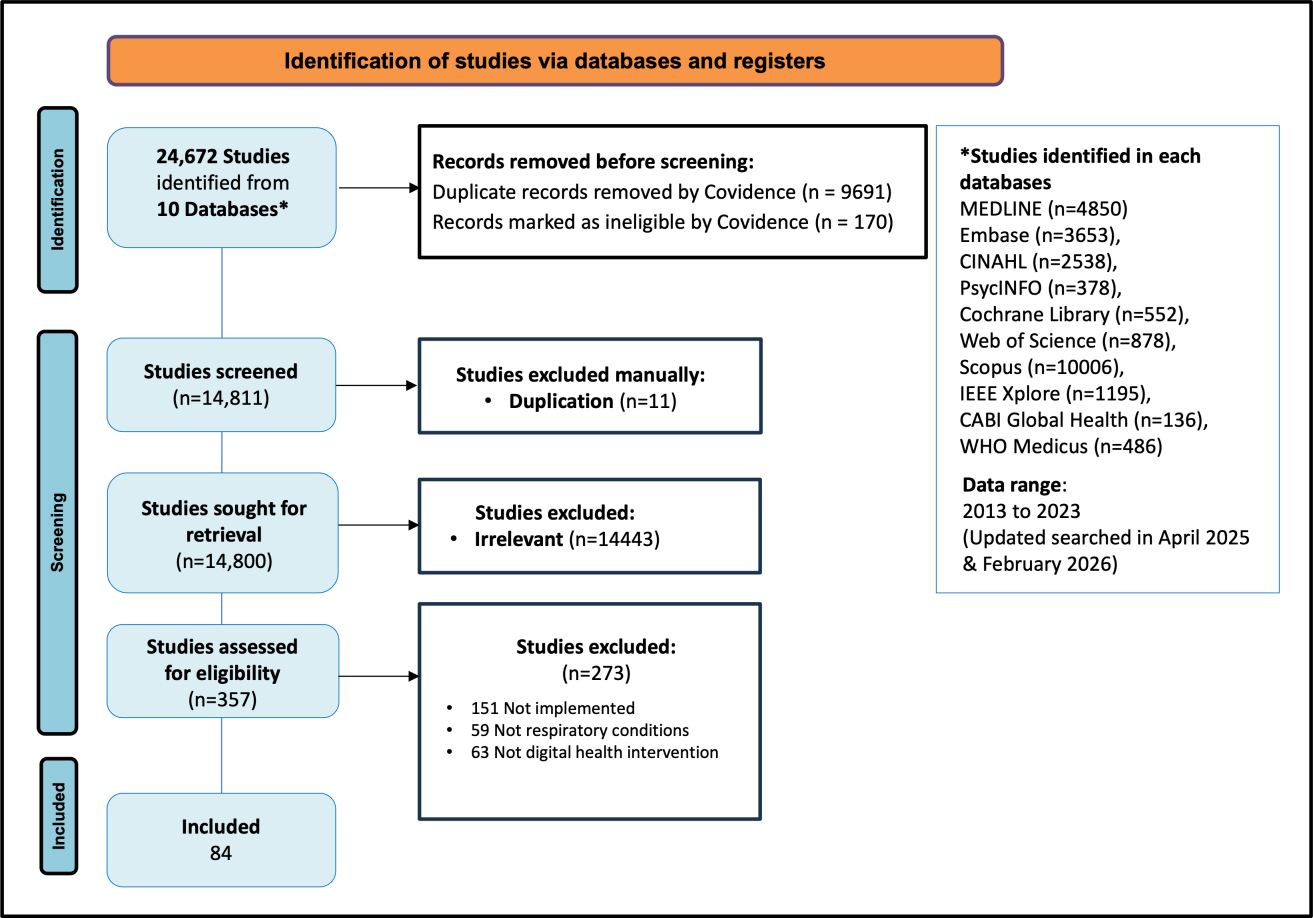
PRISMA-ScR (Preferred Reporting Items for Systematic Reviews and Meta-Analyses extension for scoping reviews) flow diagram.

Figure 2 is the PRISMA-ScR flow diagram. A total of 24,672 records were identified. After removal of duplicates, the titles and abstracts of 14,800 records were screened. Of these, 14,443 records were excluded, primarily because they focused exclusively on COVID-19 without examining an underlying respiratory condition (eg, chronic obstructive pulmonary disease [COPD] or asthma), or because they were conference abstracts, systematic reviews, or scoping reviews. Reviews were retained at the search stage rather than filtered automatically to ensure that conclusions of relevant reviews could be considered when interpreting and comparing results.

A total of 357 full-text articles were assessed for eligibility. Following full-text screening, 273 articles were excluded. The most common reasons for exclusion of full-text papers were lack of implementation in routine care (n=151; 55%), no inclusion of respiratory patients (n=59, 22%), or initiatives focused on administrative systems for management, or clinicians rather than providing patient care (n=63, 23%). A total of 84 studies [32-115] met the eligibility criteria and were included in the review.

### Study Characteristics (Objective 1)

Characteristics of the 84 included digital respiratory initiatives are described below, summarized in Table 2, and detailed in Multimedia Appendix 3 [32-115].

**Table 2.** Characteristics of the included studies.

Characteristics of the Included Program (n=84)		
Conditions	No of program (n=84), n (%)	
Asthma, asthma, and AR^a^	18 (21)	
COPD^b^, COPD and ILD^c^, COPD and HF^d^	23 (27)	
Cystic fibrosis	6 (7)	
Lung cancer	8 (10)	
Lung transplant	3 (4)	
Sleep-related breathing disorders	3 (4)	
Tuberculosis (TB)/HIV	10 (12)	
Multiple respiratory conditions	13 (15)	
Year		
Pre–COVID-19	47 (56)	
During COVID-19	32 (38)	
Post–COVID-19	5 (6)	
Participant age		
Adult (16 years or older)	61 (73)	
Pediatric (younger than 16 years)	14 (17)	
All ages	9 (11)	
Countries		
High-income countries (IHCs)	71 (85)	
Low and middle-income countries (LMICs)	7 (8)	
Rural, low-resourced regions	6 (7)	
Implementation scale		
Country	20 (24)	
Regional	33 (39)	
Service	31 (37)	

a AR: allergic rhinitis.

b COPD: chronic obstructive pulmonary disease.

c ILD: interstitial lung disease.

d HF: heart failure.

### Participants

The initiatives provided care services for pediatric (n=14; 17%) and adult (n=61; 73%) patients, or both (n=9; 11%). They mostly targeted specific respiratory conditions, but 15% (13/84) included multiple conditions. Age ranges reflected different disease profiles, with interventions for asthma [32-49] and cystic fibrosis including children [73-78]; while COPD [50-72], lung cancer [79-86], lung transplant [87-89], and sleep-related breathing disorders targeted older adults [90-92]. Tuberculosis or multiple respiratory conditions involved all age groups [93-115].

### Interventions

**Figure 3.**
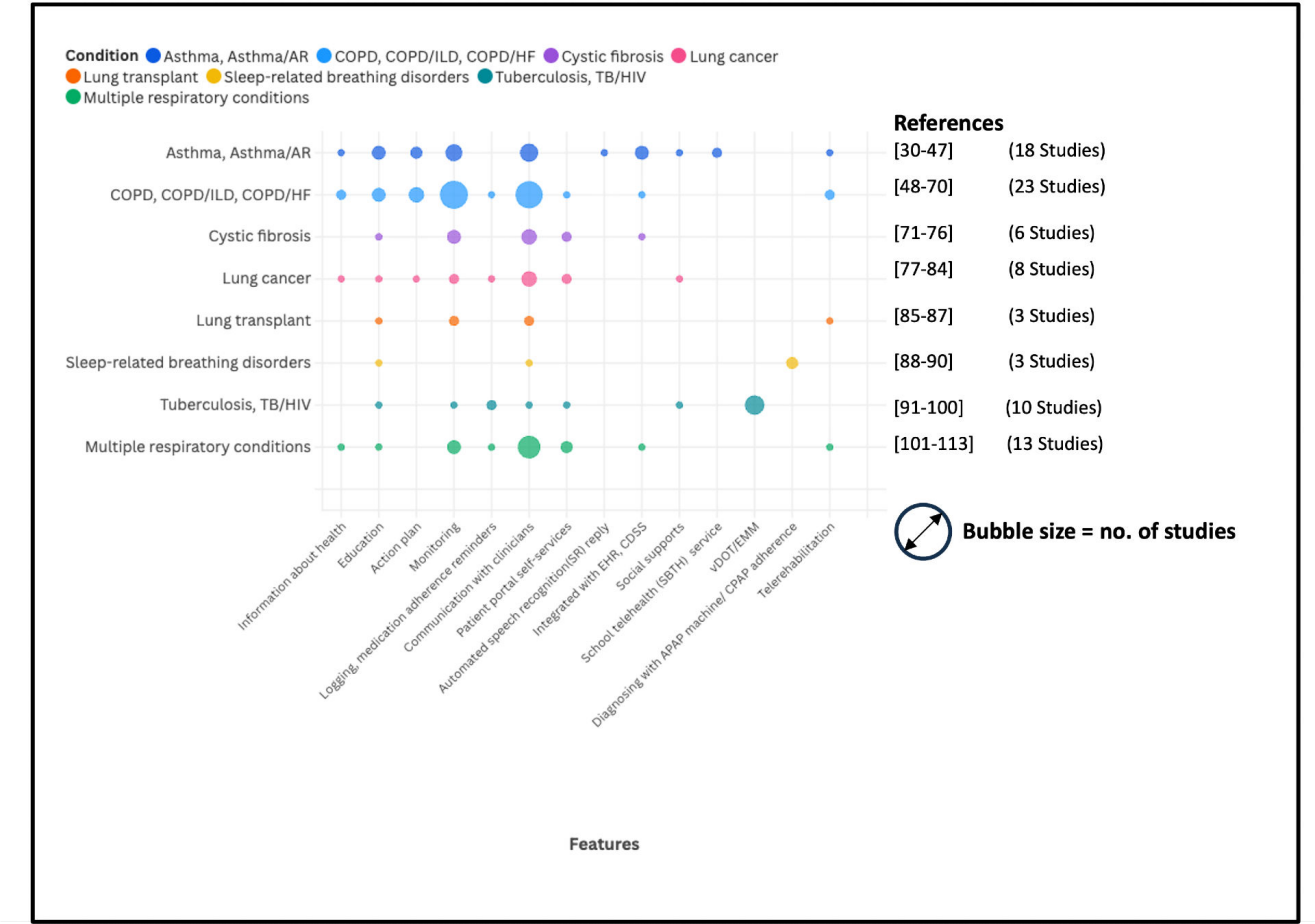
Bubble plot of the technology features and their associated respiratory conditions [32-115]. APAP: automatic positive airway pressure; AR: allergic rhinitis; CDSS: clinical decision support system; COPD: chronic obstructive pulmonary disease; CPAP: continuous positive airway pressure; EHR: electronic health record; HF: heart failure; ILD: interstitial lung disease; EMM: electronic medication monitor; vDOT: video directly observed therapy.

Features of the mobile apps, digital platforms, and monitoring devices are shown in Figure 3.

Most commonly, clinicians used an app, either alone or connected with multiple monitoring devices (n=36; 42%), videoconference, SMS, phone communication features for health coaching, follow-up consultations, discussing action plans, triage, and escalating care to other providers (n=48; 57%). Four specific categories of applications were identified:

1. Video directly observed therapy for remote monitoring of adherence to tuberculosis medication [93,95-98,101,102].
2. Diagnosis of sleep apnea and home monitoring of continuous positive airway pressure therapy [90-92].
3. Virtual rehabilitation is used to provide remote exercise training for respiratory conditions at home or community settings [46,50,68,89,111].
4. School-based telemedicine services to provide care for children with asthma in rural areas [37,41].

### Setting for the Technology Service

Most initiatives (n=64; 76%) were implemented within individual services (hospitals, GP clinics, and community centers) or regional clusters. Country-wide initiatives (n=20; 24%) included one application that was deployed in 22 countries. Of the 84 initiatives, most were deployed outside the EU region, including 36% (n=30) from the United States and 32% (n=27) from other countries such as Australia, Japan, Canada, China, India, Russia, South Africa, South Korea, and Singapore. Only 32% (n=27) originated from the EU.

### Methodology

Of the 84 studies, 81% (n=68) [33,34,36-39,41-46,49,50,52,54,56-72,80,81,84-91,93-107,111-115] were service evaluations using pre- and poststudy designs, longitudinal observational, interrupted time-series, participatory action, or case study methodology, 19% (n=16) [32,35,40,47,48,51,53,55,73,79,82,83,92,108-110] studies were a narrative summary of their implementation experiences and learning. Overall, 38% (n=32) [37,42,43,46,47,51,56-60,64,67-71,73,75-77,84-86,91,94-97,99,100,105] used interviews with patient and professional stakeholders to explore the implementation process.

### Outcomes

We did not synthesize outcomes or summarize effectiveness, as scoping review methodology is designed to map the available evidence rather than evaluate study quality or determine effectiveness. Overall, 50 studies reported clinical outcomes [32-34,36,39,45-54,56,57,59,61-65,71,72,74,77,78,81,87,89-96,98,99,101-106,109-111,113-115], with each study assessing between one and four outcomes. Of these, health care service quality-related outcomes were reported in more than half of the initiatives (n=35; 42%), followed by prescribing or treatment adherence (n=18; 21%), and health-related outcomes, including respiratory-specific outcomes such as disease control and exacerbations, quality of life, and the mental health benefits (n=6; 7%).

Two initiatives used real-world observational data from an app to explore correlations between treatment usage and asthma control [49] or risk of needing care escalation [52].

### Use of Implementation Theories, Models, and Frameworks (TMFs; Objective 2)

**Figure 4.**
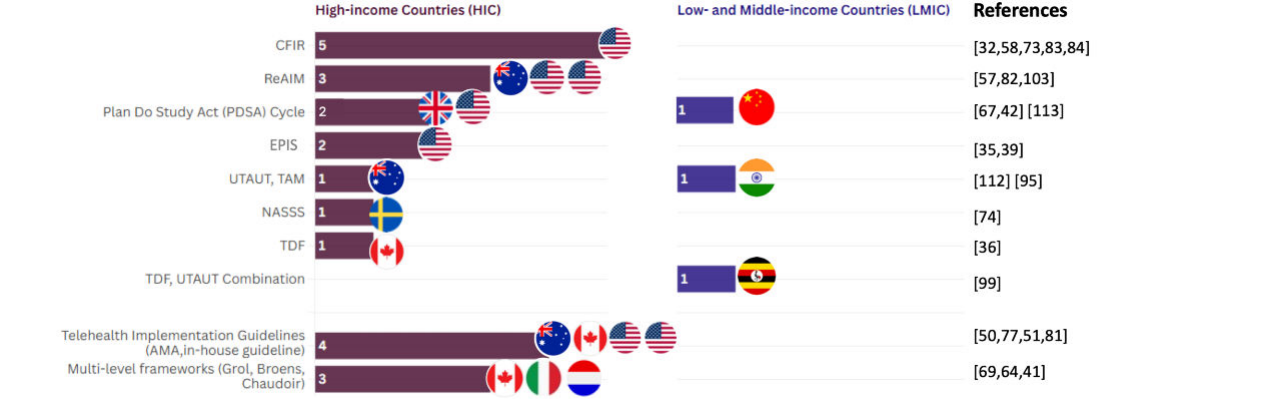
Implementation theories, models, and frameworks (TMFs) used by the high-income and low middle-income countries [34,37,38,41,43,44,52,53,59,60,66,69,71,75,76,79,83-86,97,101,105,114,115]. AMA: American Medical Association; CFIR: Consolidated Framework for Implementation Research; EPIS: Exploration, Preparation, Implementation, and Sustainment framework; NASSS: Nonadoption, abandonment, scale-up, spread, and sustainability framework; ReAIM: Reach, Effectiveness, Adoption, Implementation, and Maintenance framework; TAM: Technology Acceptance Model; TDF: Theoretical Domains Framework; TMF: Theories, Models, and Framework; UTAUT: Unified Theory of Acceptance and Use of Technology.

Of the 84 initiatives, 30% (n=25; and all the 18 country-level evaluations) used one or more TMF to guide development or evaluation (Figure 4).

The remaining 70% (n=59) of studies did not report using an implementation framework or describe their approach to initiative development. The most commonly used frameworks were CFIR (n=5; 20%) [34,60,75,85,86], RE-AIM (n=3; 12%) [59,84,105], and the plan-do-study-act (PDSA) cycle (n=3; 12%) [44,69,115]. Of the initiatives that used TMF, three were from the LMICs (China, India, Uganda). Seven service- and regional-level initiatives used a range of guidelines and multilevel models [43,52,53,66,71,79,83] to observe the digitization process. Typically, they focused on the benefits of an agile approach that incorporated feedback from patients and clinical staff.

### Common Implementation Domains

Of the 7 interconnected implementation domains from the NASSS, 6 were addressed frequently; they were “condition complexities,” “technology,” “value proposition,” “adopters,” “organization,” and “wider system” (Table 3). Only “embedding and adoption over time” was not explicitly reported in the included studies, though the need to incorporate long-term monitoring was mentioned.

**Table 3.** Common implementation domains.

Commonly reported implementation domains (n=84)	n (%)
Technology	
Integration (vendor partnerships, use of existing infrastructure, and interoperability)	36 (42.9)
System accuracy and reliability, security and privacy, confidentiality	11 (13.4)
User-friendly interface and features	6 (7.1)
Development supported by evidence	2 (2.4)
Value proposition	
Values to end users (cost, COVID-19 emergency, and service advancement)	29 (35.4)
Adopters	
Acceptability and satisfaction	38 (45.2)
Adoption, usability, and adherence rate	39 (47.6)
Co-develop with end users	13 (15.9)
Motivation, perceptions from end users	12 (14.6)
Technical support and devices to patients	11 (13.4)
Self-efficacy, knowledge	5 (6.1)
Identify early adopters	4 (4.9)
Staff willingness and organizational readiness to change	4 (4.9)
Trust from end users	2 (2.4)
Organization	
Staff training, supports, and extra workload due to implementation	26 (31)
Develop interprofessional team and workflow	26 (31)
Use opinion leaders, subject matter expertise, advisory boards, workgroups	10 (12.2)
Procurement processes, supply chain, device logistics	3 (3.7)
Wider system	
Reimbursement, insurance, and funding	16 (19)
Policy, social benefits, guideline compliance, “tech push” as driver for implementation	14 (17.1)

Within the widely reported domain of “condition complexities,” examples were both organizational (the rapid transition and unprecedented strain on health care systems of the COVID-19 pandemic [87] as well as incomplete registries in lung cancer [82,84]). Features of specific clinical contexts included multimorbidity in COPD [68], poor adherence in asthma [33,34,42], tuberculosis [93-102], and obstructive sleep apnea [90-92], as well as the high degree of disciplined self-care required in cystic fibrosis [76]. Technology domains such as choosing in-house development or a proprietary solution, or integration with the electronic health record were mostly considered in the development phases. Value proposition domains were described as the incentive for launching the initiative. Adoption and organizational factors were influenced by inter-relationships, communication, and culture. Opinion leaders and subject expertise could drive implementation. Wider system domains included both internal and external service goals, political or policy aims, intervention costs (money; time), and funding (grants; reimbursement).

### Implementation Barriers and Facilitators (Objective 3)

**Figure 5.**
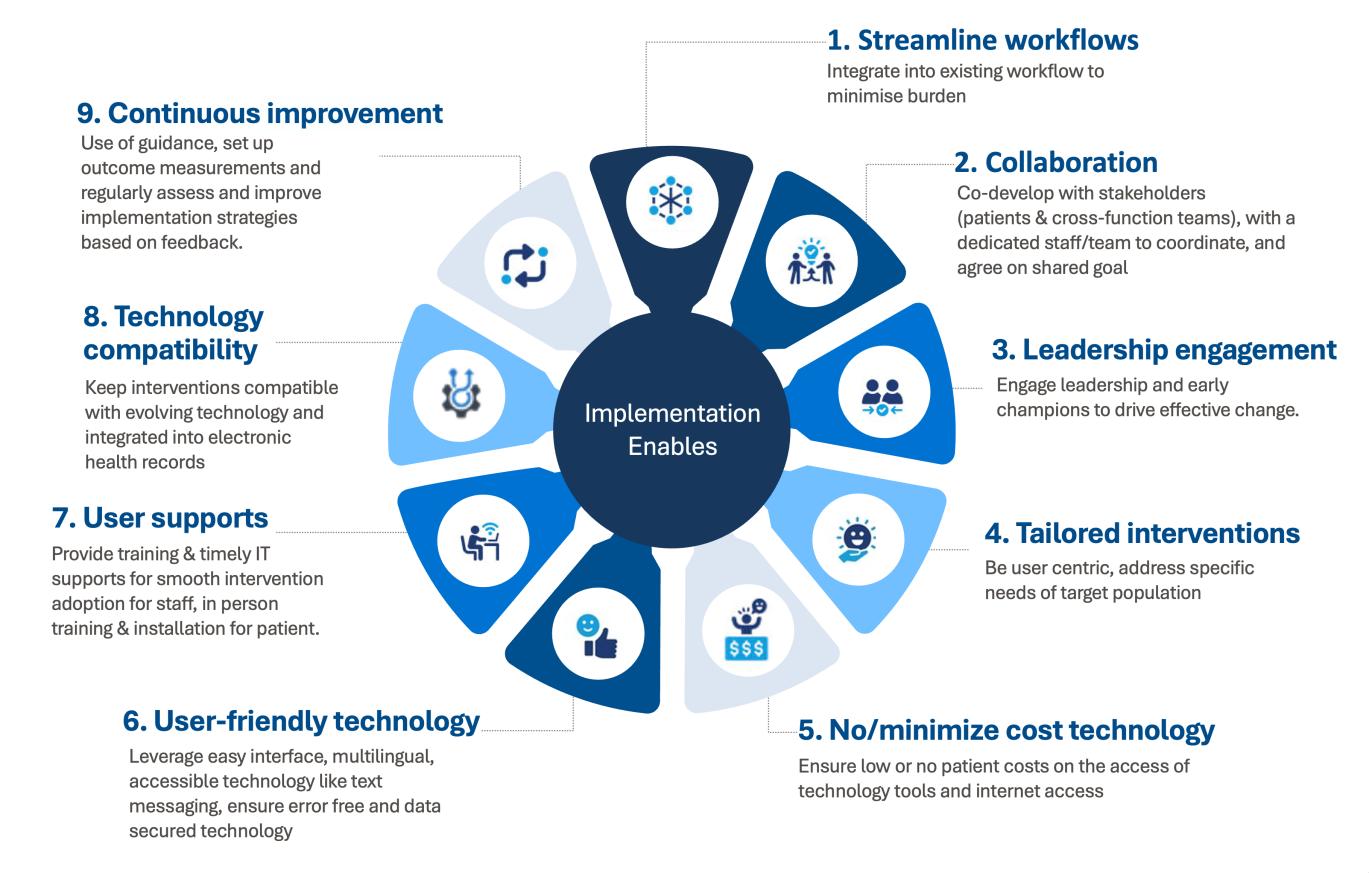
Facilitators for effective implementation.

More than half (n=54; 64%) of the studies reported barriers and/or enablers to implementing digital health initiatives in routine care [33-43,46,47,50-52,55,57-60,64-69,71,73,75,76,79,80,82,84-89,92,93,95-97,100-102,106-108,110,113,114]. These are summarized in Figures5  6; with details in Multimedia Appendix 3.

**Figure 6.**
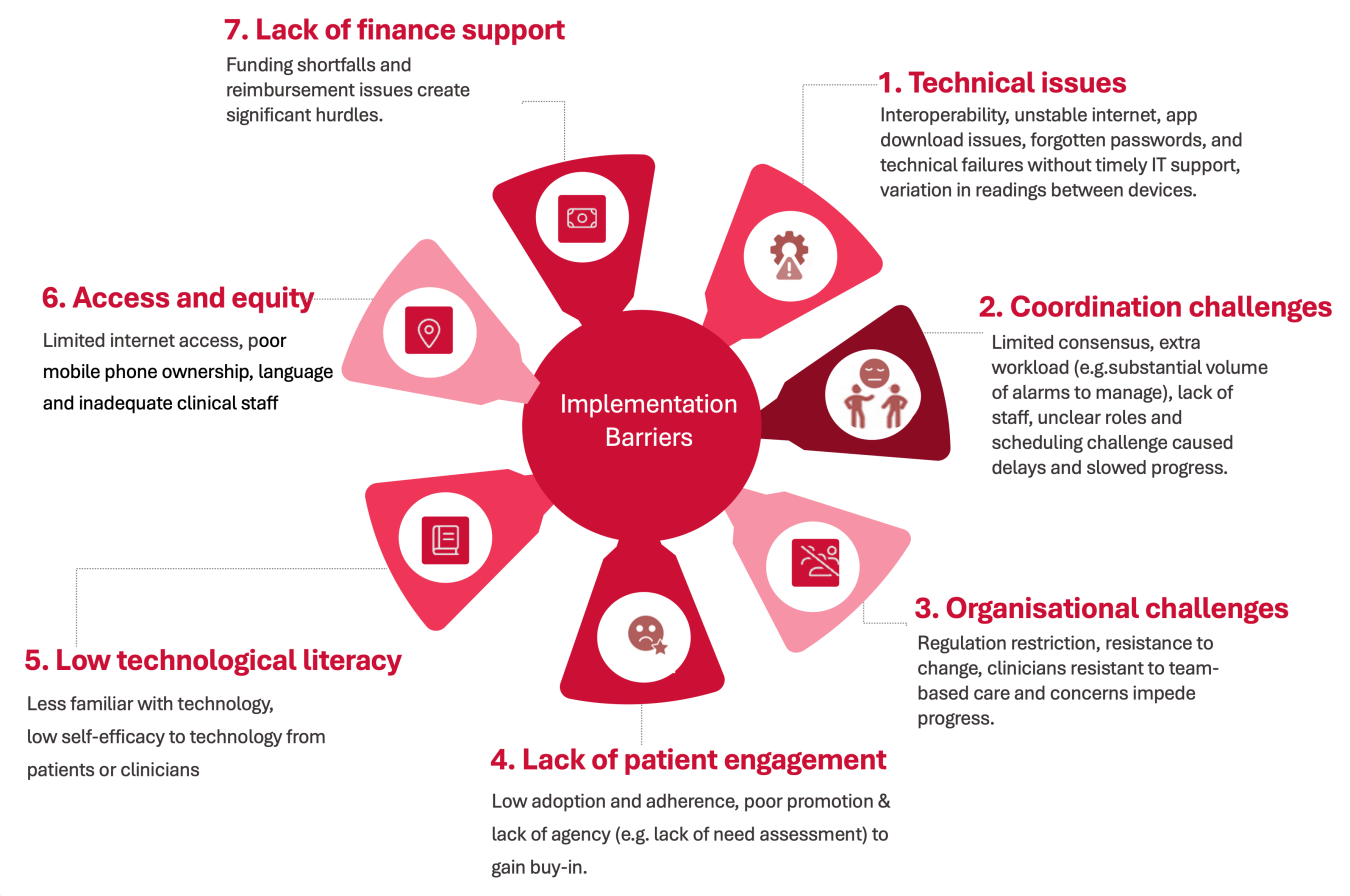
Barriers for effective implementation.

Key issues included:

1. Technical barriers (poor integration with existing technology; software installation problems; password barriers) could be reduced by error-free, user-friendly interfaces, tailored to the needs of the target population and offering easy access to timely IT support.
2. Coordination challenges (implementation teams with unclear roles; difficulty scheduling meetings) could be mitigated by facilitating co-development from the outset (common goals; clear expectations) and allowing sufficient committed staff time.
3. Streamlining processes and integrating into existing workflow to avoid additional workload (eg, duplicate data entry) reduced resistance to adopting new ways of working.
4. Engaged leadership and local champions helped convince staff of the value of the innovation and overcome reservations (eg, about third-party providers of systems; or risk to valued interpersonal contact).
5. Optimizing patient engagement by raising awareness, promoting buy-in, and improving technological literacy by using co-design, developing innovations that were patient-friendly, multilingual, addressed actual needs, and used data-secured technology.
6. Removing structural barriers by minimizing cost to the patient (free devices and internet access), thereby reducing unaffordability and the risk of increasing inequities.
7. Funding models that did not reimburse the full costs of a technology-based service (eg, required upgrades of existing legacy systems to maintain compatibility, necessitated multidisciplinary support staff costs) created significant hurdles for clinicians to adopt and sustain the initiative.

Studies reported that the COVID-19 pandemic was a key facilitator in eliminating some previous barriers (eg, additional funding streams and coordination support) as the traditional care model was changed rapidly to digital. Attitudes shifted as both patients and providers recognized the need for social distancing and remote care delivery overriding preferences for valued direct patient interactions.

### Insights (Objective 4)

#### Health Inequity

Digital health initiatives that incorporated remote monitoring and brought services closer to the point of need such as providing remote consultations within schools for students with respiratory conditions would be an effective strategy for improving access to care, but initiatives from LMICs [82,93-97,100,101,113,115], rural regions, and low-income populations [37,41,52,58,85,112] faced significant barriers such as poor transportation, no insurance, poor mobile phone coverage, and health care provider shortage. Tailored interventions and language support facilitated the needs of diverse subpopulations.

#### Patient and Professional Relationships

Remote consulting could reduce clinicians’ workloads but raises concerns about dehumanization and reduced patient engagement compared to face-to-face interactions [34]. Remote pediatric consultations struggled to involve the child (though easier if the child already knew the clinician), and even parents were less active than in face-to-face appointments [44,46]. Building rapport was hindered by clinicians’ difficulty sensing body language and responding to emotional cues [43,58,64,86]. Initiatives with 2-way messaging improved access, but ensuring timely responses was difficult [43]. Practical challenges included difficulty checking objective signs [70,73,75,88]. Retaining the option of in-person consultations was deemed safe and respectful for individual preferences [46,68].

#### Patient Journey

Digital initiatives allowed patients to receive support in their own homes and could improve continuity of care [47,53,107], empower self-care, and ensure that care plans reflected individual needs and preferences [55,81], while reducing barriers of travel time and costs [34,67,91,108,111]. Connected care models, such as a platform connecting multiple monitoring devices or enabling data sharing with specialists across departments, helped to coordinate support, simplify the patient journey, and improve access to multiple specialists [73,77].

#### Environmental Impact

Remote monitoring and consultation minimized travel [42,58,95,112], especially in rural areas, potentially improving attendance at pulmonary rehabilitation [111]. By decreasing reliance on carbon-intensive travel for health care appointments, digital approaches supported both environmental sustainability and economic efficiency.

**Figure 7.**
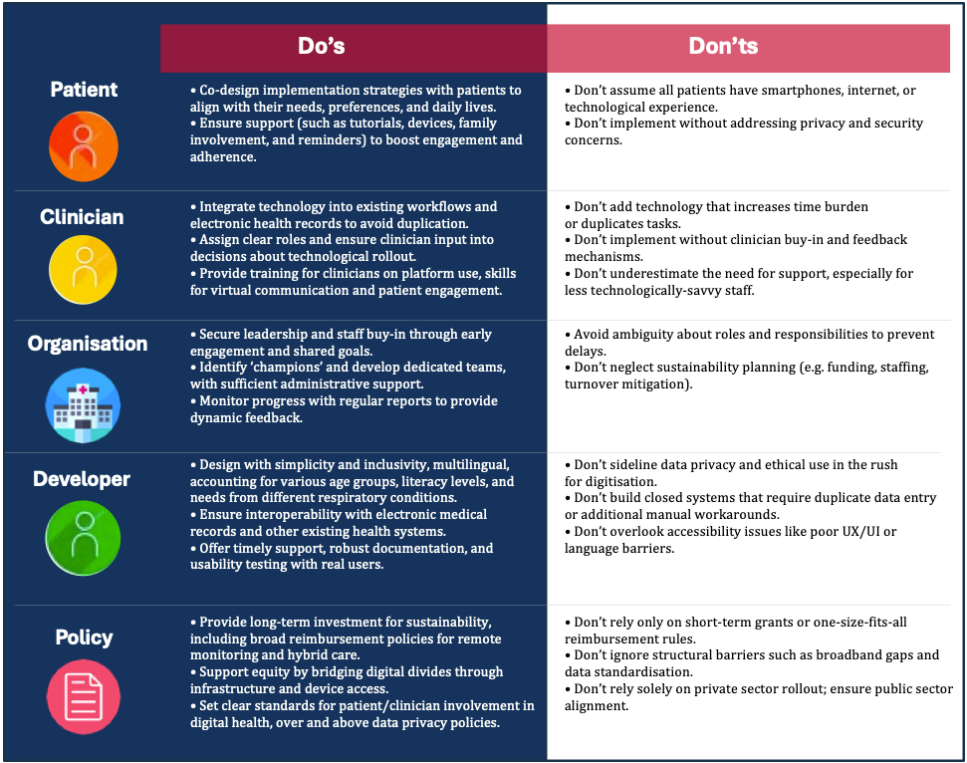
Do’s and don’ts for implementation. UI: user interface; UX: user experience.

We have summarized the do’s and don’ts for implementation in Figure 7.

## Discussion

### Summary of Findings

This review provides an overview of evidence on the implementation of digital technologies from 84 digital respiratory care initiatives across diverse populations and settings. The most targeted population was adult patients with a range of respiratory conditions. The initiatives used apps, digital platforms, and smart devices to enable remote monitoring and facilitate communication with clinicians for care planning and management. Health care service quality, prescribing or treatment adherence, and respiratory-specific clinical outcomes were the most reported outcome measurements. Fewer than a third of initiatives, typically national initiatives in high-income countries, used TMFs such as CFIR, RE-AIM, and PDSA cycles to address condition complexities, technology, value proposition, adopters, organization, and wider system factors. Barriers to implementation included limited technical integration, low patient engagement, funding shortfalls, and organizational challenges, mitigated by early involvement of stakeholders, leadership and champions, user-friendly design, and providing training and support.

### Interpretation and Implications in Relation to Published Literatures

#### Use of Implementation Frameworks

Fewer than a third of the studies used a TMF to guide the implementation and evaluation of their initiative—and those that did were typically country-level initiatives in high-income settings (Figure 4). The comprehensive approaches to development and evaluation embodied by the CFIR and the RE-AIM frameworks may be perceived as resource-intensive, or too complex for local initiatives, or not adapted to the specific policies, infrastructure, and social-cultural adjustments required in LMICs [116]. However, it is notable that the least reported domains (Table 3) are “organizational” and the “wider context;” they emerge as key barriers and enablers, suggesting that a structured approach to digital implementation could be helpful in adapting to diverse clinical, health care systems and socioeconomic contexts.

#### Complexities in Implementation

Clinical complexity challenged the implementation of digital health care for respiratory conditions. For example, multimorbidity is common in COPD, and initiatives required technology developers to integrate systems for multiple conditions in often deprived social contexts whilst providing error-free technology that is user-friendly and compliant with global data privacy regulations [117]. The organizational enablers and barriers resonated with those found in a recent survey of stakeholders who facilitated innovation adoption across 18 UK National Health Service sites [118]. Ensuring timely technical support and involving cross-functional teams and patients early in the design phase were critical (but complex) tasks requiring strong leadership to align stakeholders and manage resources effectively [119]. Additionally, the challenges and enablers were dynamic; initial barriers like staff resistance due to perceived workload could evolve into facilitators as confidence in the intervention grew [120]. The COVID-19 pandemic accelerated adoption by easing regulations and reimbursement barriers, shifting preferences toward remote care [5].

Complexity in implementation reflects the multiple stakeholders involved in developing, using, and maintaining digital care and the need to align organizational change with existing workflows and routines [121,122]^.^

#### Future Challenges for Implementation

Two key challenges that remain under-explored are avoiding increasing inequity and considering environmental impacts. Initiatives prioritized clinical outcomes and service outcomes over assessing differential access, usability, or benefits across diverse socioeconomic, geographic, and demographic groups.

#### Digital Divide

A substantial number of studies showed that, despite their promise, digital health technologies can inadvertently widen existing health inequities—“the digital divide”—particularly in LMICs, rural areas with poor infrastructure, or other resource-constrained settings [123]. About one in five studies in this review highlighted inequity as a concern [37,41,52,58,82,85,93-97,100,101,112,113,115]. Disparities in broadband availability, digital literacy, and culturally responsive technology design disproportionately burden underserved groups, increasing uneven uptake and consequently exacerbating differential health outcomes [124]. Digital-only interventions heighten vulnerability in these populations and may entrench, rather than alleviate, socioeconomic barriers to care [125]. In response, care-coordination models that provide structured, personalized support, encompassing routine screening for digital-health readiness, proactive patient navigation (eg, including community health workers), and coordinated cross-sector referrals, offer plausible countermeasures by embedding digital tools within human-facilitated, inclusive workflows [126]. However, realizing the benefits of these equity-first hybrid models requires explicit operational definitions and clear delineation of responsibilities between technological components and coordinating staff, alongside sustained solutions for root-cause constraints such as reliable access to devices (eg, mobile phones, wearables, and AI), affordable and stable connectivity [127].

#### Environmental Sustainability

Within the studies in this review, 5 considered environmental sustainability to be an important benefit of the technology initiative [42,58,95,111,112]. Remote care is often framed as a means of reducing carbon emissions and generating economic benefits [128]. However, the energy-intensive infrastructure required to develop and operate AI systems raises concerns about increased energy consumption, making the long-term net carbon impact on health care uncertain [129]. Training large-scale AI models, such as deep neural networks and generative models, demands substantial computational power [130]. In the meanwhile, AI combined with internet of things and the things network smart-city sensors offers new ways to address environmental challenges, including disaster forecasting, climate modeling, and operational energy optimization. Examples include sentiment analysis to shape environmental messaging, reinforcement learning to optimize water and energy use, and AI simulations to evaluate conservation strategies under varied ecological conditions [131]. Furthermore, the emerging concept of “Green AI” calls for quantifying embodied carbon and operational energy use across the full lifecycle of AI infrastructure, from construction to decommissioning data centers, together with associated Scope 1‐3 (direct and indirect) supply-chain emissions, to support meaningful carbon reduction efforts [132,133]. However, deploying AI in routine respiratory care by adding health care-specific supply chains (eg, inhaler recycling pathways) can contribute to an overall beneficial footprint [134]. Further research is needed to develop pragmatic key performance indicators and measurement frameworks that capture both carbon impacts and health benefits.

#### Trust

Reduced personal interaction in remote consultations raises trust concerns. Strong patient-clinician relationships require confidence in technology, clear motives, and empathetic responses [135]. Despite adoption being widely discussed, “trust from end users” was only mentioned in 2% of the included studies, suggesting the need to increase the focus on this core attribute (Table 3). As generative AI becomes increasingly integrated into daily life, evaluating its perceived empathy and trustworthiness in health care is increasingly crucial. Moreover, perceptions of algorithmic bias, data security, and transparency can significantly influence whether patients feel comfortable engaging with AI-supported care [136,137]. Establishing robust guidelines for how AI systems communicate with patients, who is responsible when things go wrong, and how humans supervise AI decisions could help reduce worries about trust, safety, and reliability [138,139]. Ultimately, systematically assessing how different populations experience trust in AI-enabled health care will be essential for equitable and sustainable implementation.

### Strengths and Limitations

We used standard methodology PRISMA-ScR and PRISMA-S methodology, while considering the reporting items suggested by Arksey and O’Malley [27] to ensure a transparent and systematic approach to this mixed-methods scoping review [23]. The collaborative process enabled input throughout the review from a multidisciplinary global team with academic and clinical interest in digital respiratory health.

There were some limitations. Despite the use of broad search terms, poor indexing of implementation research may have excluded relevant studies. Gray literature was omitted due to concerns regarding the methodological quality and limited resources. We set a 10-year search time limit to ensure our review reflected current technology and its implementation, but may have missed valuable lessons from older, well-established initiatives. We included studies from both LMICs and high-income countries to ensure global representation; however, the diverse health care contexts meant the included studies were heterogeneous. To facilitate contextual interpretation, we grouped the findings into low- and high-income countries based on the World Bank’s income classification. Most of the initiatives were deployed in non-EU countries, where barriers, such as regulatory requirements and funding structures, were substantially different from those in the EU or low-resource settings. Nevertheless, this broad geographical representation provided valuable insight into common barriers and facilitators at an international level, and our focus on TMFs is a strength, as a comprehensive framework can support the adaptation required to transfer ideas between diverse health care systems.

Our scoping review provides an overview of digital respiratory initiatives rather than evaluating their effectiveness. Our analysis is limited by reported data, and could not detect unreported factors, but key implementation challenges and facilitators were highlighted in most papers.

### Conclusions

This scoping review provides insights into factors affecting the implementation of digital respiratory health care, summarizing 84 digital respiratory initiatives, the frameworks they used, and the barriers and facilitators to successful implementation in routine care. The findings provide a contemporary overview of the use of TMFs across LMICs and high-income countries, highlighting the value of structured implementation of TMFs in supporting adoption across diverse clinical settings, health care systems, and socioeconomic contexts. It offers an empirical basis to inform future policy statements and guidance on standardized approaches to the development, evaluation, and reporting of digital health care interventions. Meaningful engagement of multiple stakeholders throughout the project lifecycle is essential to ensure alignment with patient needs and organizational workflows, thereby minimizing additional workload and safeguarding time for direct patient care. Addressing persistent and emerging challenges, including the digital divide, environmental sustainability, and the establishment of trust between technologies such as AI, patients, and clinicians, will be essential to support effective and scalable delivery of digital respiratory health care services.

## Supplementary material

10.2196/88325 Multimedia Appendix 1 Detailed search strategy.

10.2196/88325 Multimedia Appendix 2 Detailed criteria and definitions.

10.2196/88325 Multimedia Appendix 3 Full data extraction table.

10.2196/88325 Checklist 1 PRISMA-ScR reporting checklist.

## Abbreviations

AI: artificial intelligence
CFIR: Consolidated Framework for Implementation Research
COPD: chronic obstructive pulmonary disease
CRC: Clinical Research Collaboration
EPIS: exploration, preparation, implementation, and sustainment
ERS: European Respiratory Society
EU: European Union
LMIC: low and middle income countries
NASSS: Nonadaptation, Abandonment, and Challenges to the Scale-up, Spread, and Sustainability of Health and Care Technologies
PDSA: plan-do-study-act
PRISMA: Preferred Reporting Items for Systematic Reviews and Meta-Analyses
PRISMA-ScR: Preferred Reporting Items for Systematic Reviews and Meta-Analyses extension for scoping reviews
RE-AIM: reach, effectiveness, adoption, implementation, and maintenance
TAM: Technology Acceptance Model
TMF: Theories, Models, and Framework
UTAUT: Unified Theory of Acceptance and Use of Technology
WHO: World Health Organization
