# Supplementary material for: Implementing Digital Respiratory Technologies for People With Respiratory Conditions: Scoping Review
Source: J Med Internet Res. 2026 Jun 16;28:e88325. doi: 10.2196/88325 (PMC13271594; doi:10.2196/88325)
Supplement: Multimedia Appendix 1 [file jmir-v28-e88325-s001.docx]

| **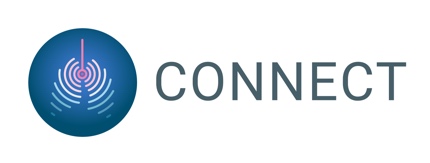** | **Implementing digital respiratory technologies for people with respiratory conditions: a systematic scoping review**  **(Short title: Digital respiratory health implementation – systematic scoping review)** |
| --- | --- |

*Io Chi Yan Hui, Kathleena Condon, Shailesh Kolekar, Nicola J Roberts, Katherina Bernadette Sreter, Sami O. Simons, Carlos Figueiredo, Zoe McKeough, Hani Salim, Aleksandra Gawlik-Lipinski, Apolline Gonsard, Ayşe Önal Aral, Anna Vanoverschelde, Matthew Armstrong, Dario Kohlbrenner, Cátia Paixão, Patrick Stafler, Efthymia Papadopoulou, Adrian Paul Rabe, Milan Mohammad, Izolde Bouloukaki, Shirley Quach, Georgios Kaltsakas, Kate Loveys, Tonje Reier-Nilsen, Anthony Paulo Sunjaya, Paul David Robinson, Michaela Senek, Amy Hai Yan Chan, Hilary Pinnock*

**Multimedia Appendix S1 for detailed search strategy**

**Ovid MEDLINE(R)**

**Keywords and subheading**

1 (Telemedicine or telecare or telehealth).ti,ab,kw.

2 (ehealth or e-health or mhealth or m-health).ti,ab,kw.

3 digital transformat*.ti,ab,kw.

4 digital strateg*.ti,ab,kw.

5 digital polic*.ti,ab,kw.

6 digital health.ti,ab,kw.

7 (smartphone* or smart-phone*).ti,ab,kw.

8 ((cell* or mobile*) adj3 phone*).ti,ab,kw.

9 (mobile adj3 tablet$1).ti,ab,kw.

10 (portable adj1 device$1).ti,ab,kw.

11 (portable adj1 sensor$1).ti,ab,kw.

12 gadget$1.ti,ab,kw.

13 (smart adj1 device$1).ti,ab,kw.

14 (smart adj1 sensor$1).ti,ab,kw.

15 (smart inhaler* or digital inhaler* or "Electronic Monitoring Device*").ti,ab,kw.

16 ((smart adj3 peak flow meter*) or spirometer*).ti,ab,kw.

17 (handheld* or hand-held*).ti,ab,kw.

18 wearable*.ti,ab,kw.

19 ("In vitro diagnostic*" adj3 device*).ti,ab,kw.

20 (automat* adj3 (phone* or telephone* or call* or system*)).ti,ab,kw.

21 (app* adj3 (smartphone* or smart-phone or mobile* or phone* or tablet* or computer*)).ti,ab,kw.

22 (internet* or web*).ti,ab,kw.

23 (sms or mms).ti,ab,kw.

24 ((text* or short*) adj3 messag*).ti,ab,kw.

25 Texting.ti,ab,kw.

26 ((electronic* adj3 (patient* or health or medic*) adj3 record*) or "EPR" or "EHR" or "EMR").ti,ab,kw.

27 (reminder adj3 (text* or system* or messag*)).ti,ab,kw.

28 (alert* adj (system or device)).ti,ab,kw.

29 ((internet adj1 of adj1 thing*) or "iot").ti,ab,kw.

30 ("virtual reality" or "VR").ti,ab,kw.

31 "augmented reality".ti,ab,kw.

32 "mixed reality".ti,ab,kw.

33 "eXtended reality".ti,ab,kw.

34 "artificial intelligence".ti,ab,kw.

35 "machine learning".ti,ab,kw.

36 "interactive voice response".ti,ab,kw.

37 chatbot.ti,ab,kw.

38 "digital twin".ti,ab,kw.

39 "blockchain".ti,ab,kw.

40 respir*.ti,kw.

41 (chronic$ adj3 (lung$ or respiratory$ or pulmonary$)).ti,kw.

42 ((chronic* or obstruct*) adj3 (pulmonary or lung* or airway* or airflow* or bronch* or respirat*)).ti,kw.

43 (interstitial$ adj3 (lung$ or disease$ or pneumon$)).ti,kw.

44 ((pulmonary$ or lung$ or alveoli$) adj3 (fibros$ or fibrot$)).ti,kw.

45 (idiopathic and pulmonary and fibrosis).ti,kw.

46 ((pulmonary$ or lung$) adj3 (sarcoid$ or granulom$)).ti,kw.

47 (asthma* or wheez*).ti,kw.

48 emphysema*.ti,kw.

49 (COPD or COAD or COBD or AECOPD or AECB).ti,kw.

50 Bronchi*.ti,kw.

51 (cystic* adj3 fibros*).ti,kw.

52 (interstitial* adj3 (lung* or disease* or pneumon*)).ti,kw.

53 ((pulmonary* or lung* or alveoli*) adj3 (fibros* or fibrot*)).ti,kw.

54 (pneumoconiosis or silicosis).ti,kw.

55 (pulmonary adj3 eosinophi*).ti,kw.

56 (pulmonary adj2 hypertensi*).ti,kw.

57 (pulmonary adj3 sarcoid*).ti,kw.

58 sleep adj3 apnoea*.ti,kw.

59 ((lung* or pulmonary) adj3 (cancer or tumor or tumour)).ti,kw.

60 (tuberculosis or "TB").ti,kw.

61 (extrinsic adj3 allergic adj3 alveolitis).ti,kw.

62 (pneumonia or "respiratory infection").ti,kw.

63 (COVID or nCoV or SARS or MERS).ti,kw.

64 (real-world or "real world").ti,ab,kw.

65 ((evidence-based or "evidence based") adj1 trial$1).ti,ab,kw.

66 ((evidence-based or "evidence based") adj1 stud$3).ti,ab,kw.

67 ((evidence-based or "evidence based") adj1 intervention$1).ti,ab,kw.

68 ((real-world or "real world") adj1 (trial* or stud* or research or intervention*)).ti,ab,kw.

69 ((real-world or "real world") adj1 (setting* or practice or context*)).ti,ab,kw.

70 ((routine or normal) adj1 (setting* or practice or care or context*)).ti,ab,kw.

71 implement*.ti,kw.

72 (re-aim or reaim or CFIR or "consolidated framework for implementation research" or ("NASSS" adj1 framework*) or "Normalisation Process Theory" or "Standards for Reporting Implementation Studies").ti,ab,kw.

73 adopt*.ti,kw.

73 deploy*.ti.kw

73 exp Telemedicine/

74 exp "Delivery of Health Care"/

75 exp Smartphone/

76 exp Cell Phone/

77 exp Biological Monitoring/

78 exp Fitness Trackers/

79 exp Drug Monitoring/

80 exp Computers, Handheld/

81 exp Telephone/

82 exp gamification/ or exp mobile applications/

83 exp Internet/

84 exp Text Messaging/

85 exp Electronic Health Records/

86 exp Medical Records Systems, Computerized/

87 exp Patient Portals/

88 exp Reminder Systems/

89 exp decision support systems, clinical/ or exp health information systems/ or exp integrated advanced information management systems/

90 exp "Internet of Things"/

91 exp virtual reality/

92 exp augmented reality/

93 exp artificial intelligence/ or exp machine learning/

94 exp Blockchain/

95 exp respiratory tract diseases/ or exp bronchial diseases/ or exp lung diseases/ or exp lung diseases, fungal/ or exp lung diseases, interstitial/ or exp lung diseases, obstructive/ or exp pulmonary disease, chronic obstructive/ or exp lung diseases, parasitic/ or exp pneumonia/ or exp tuberculosis, pulmonary/

96 exp Sleep Apnea Syndromes/

97 exp pneumonia, viral/ or exp covid-19/

98 exp severe acute respiratory syndrome-related coronavirus/ or exp sars-cov-2/

99 exp "diffusion of innovation"/ or exp implementation science/ or exp technology transfer/

100 exp Program Evaluation/

101 exp health plan implementation/ or exp technology assessment, biomedical/

102 1 or 2 or 3 or 4 or 5 or 6 or 7 or 8 or 9 or 10 or 11 or 12 or 13 or 14 or 15 or 16 or 17 or 18 or 19 or 20 or 21 or 22 or 23 or 24 or 25 or 26 or 27 or 28 or 29 or 30 or 31 or 32 or 33 or 34 or 35 or 36 or 37 or 38 or 39 536476

103 40 or 41 or 42 or 43 or 44 or 45 or 46 or 47 or 48 or 49 or 50 or 51 or 52 or 53 or 54 or 55 or 56 or 57 or 58 or 59 or 60 or 61 or 62 or 63

104 64 or 65 or 66 or 67 or 68 or 69 or 70 or 71 or 72

105 73 or 74 or 75 or 76 or 77 or 78 or 79 or 80 or 81 or 82 or 83 or 84 or 85 or 86 or 87 or 88 or 89 or 90 or 91 or 92 or 93 or 94 or 102

106 95 or 96 or 97 or 98 or 103

107 99 or 100 or 101 or 104

108 102 and 103 and 104

109 105 and 106 and 107

110 limit 108 to last 10 years

111 limit 109 to last 10 years

112 102 and 106 and 107

113 limit 112 to last 10 years

**CABI library -** [**https://www.cabidigitallibrary.org/**](https://www.cabidigitallibrary.org/)

Note: Only allow limited number of search terms, no subheading/MeSH terms are available.

**Technology search terms**

telemedicine OR telecare OR telehealth OR ehealth OR mhealth OR "digital health"

"smartphone app" OR internet* OR "SMS" OR "MMS" OR "text messaging" OR “cell phone” OR “”telephone”

"electronic patient record" OR "electronic health record" OR "electronic medical record"

"smart inhaler" OR "digital inhaler" OR "Electronic Monitoring Device"

"artificial intelligence" OR "handheld device" OR "wearable device"

**Respiratory condition search terms**

respir*

chronic* lung OR chronic* respiratory OR chronic* pulmonary

COVID OR nCoV OR SARS OR MERS

**Implementation search terms**

"real world" OR "routine setting" OR “routine practice" OR "routine care" OR "routine context" OR "re-aim" OR "reaim" OR "cfir" OR "consolidated framework for implementation research" OR "nasss" OR "normalisation process theory" OR "standards for reporting implementation studies"
