# Supplementary material for: Implementing Digital Respiratory Technologies for People With Respiratory Conditions: Scoping Review
Source: J Med Internet Res. 2026 Jun 16;28:e88325. doi: 10.2196/88325 (PMC13271594; doi:10.2196/88325)
Supplement: Multimedia Appendix 2 [file jmir-v28-e88325-s002.docx]

| **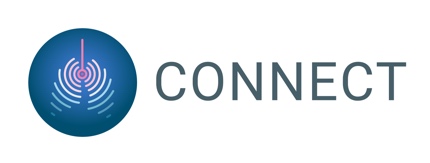** | **Implementing digital respiratory technologies for people with respiratory conditions: a systematic scoping review**  **(Short title: Digital respiratory health implementation – systematic scoping review)** |
| --- | --- |

*Io Chi Yan Hui, Kathleena Condon, Shailesh Kolekar, Nicola J Roberts, Katherina Bernadette Sreter, Sami O. Simons, Carlos Figueiredo, Zoe McKeough, Hani Salim, Aleksandra Gawlik-Lipinski, Apolline Gonsard, Ayşe Önal Aral, Anna Vanoverschelde, Matthew Armstrong, Dario Kohlbrenner, Cátia Paixão, Patrick Stafler, Efthymia Papadopoulou, Adrian Paul Rabe, Milan Mohammad, Izolde Bouloukaki, Shirley Quach, Georgios Kaltsakas, Kate Loveys, Tonje Reier-Nilsen, Anthony Paulo Sunjaya, Paul David Robinson, Michaela Senek, Amy Hai Yan Chan, Hilary Pinnock*

**Multimedia Appendix S2: Detailed criteria and definitions**

|  | **Inclusion and exclusion criteria, data range and sources of searches** |
| --- | --- |
| **Population** | Patients with any respiratory conditions (short or long-term) of all ages. |
| **Intervention** | Any digital health intervention implemented to support routine patient care. Patient care can include but is not limited to diagnosis, self-management, monitoring, medication adherence/compliance, education, psychological support, social support, remote consultation, or health professional facing interventions. For example, clinical decision supports that are designed to support delivery of care are included. |
| **Settings** | Any healthcare setting. |
| **Study focus** | Implementation studies. We recognise that these are not always clearly identified, but key features include:   - The intervention should be available to all clinically-eligible individuals (specifically not only to people who consent to the research). - Outcomes should reflect the uptake and impact of the intervention within the population (e.g. using routine data). - We have not specified a duration from implementation to the evaluation, but the focus is on studies in which an intervention is embedded for the long term in routine care. - The intervention should be delivered within the existing service – who may be upskilled for the purpose. Interventions that require additional staff/resources for day-to-day delivery are unlikely to be sustained beyond the end of the project. |
| **Languages** | Any. |
| **Date range** | 2013-2023 (10 years), updated April 2025, February 2026 |
| **Databases** | MEDLINE, EMBASE, CINAHL, PsychINFO, Cochrane Library, Web of Science Core Collection and ISI Proceedings, ScienceDirect, IEEE Xplore  LMIC focused databases: CABI Global Health, WHO Medicus databases |
| **Search terms** | See Appendix 1 which details the search terms in Medline and CABI. For the included studies, we will snowball search or contact their main authors to check if they have/ have published a qualitative result. If so, we will include their results in the review. |
| **Forward citations** | A forward search will be performed on all included studies using the International Statistical Institute Proceedings. The reference list of all eligible studies will be scrutinised to identify additional possible studies. |
| **Unpublished and in progress studies** | We will not include unpublished interventions.  We will not include conference abstracts, but will tag potentially relevant studies and check for a subsequent publication.  We will not include systematic reviews, but will tag them and check their list of included studies. |
| **Other exclusion criteria** | - Digital health interventions that do not involve patients. - Interventions with exclusively administrative purposes (e.g. workflow, appointments or triage systems). - Health professional training interventions for exclusively education purposes (e.g. online conferences, online courses). - Population level COVID-19 pandemic-management initiatives (such as contact tracing, vaccination programmes) unless the initiative is specifically focussed on people with respiratory disease (e.g. management of COVID-19 in people with COPD) |
